# A Novel Fluorescent Test Papers Based on Carbon Dots for Selective and Sensitive Detection of Cr (VI)

**DOI:** 10.3389/fchem.2020.595628

**Published:** 2020-12-01

**Authors:** Yizhou Yang, Xuemei Chen, Yangyang Wang, Miao Wu, Yinan Ma, Xudong Yang

**Affiliations:** ^1^Key Laboratory of Advanced Structural Materials, Ministry of Education, School of Materials Science and Engineering, Changchun University of Technology, Changchun, China; ^2^School of Chemical Engineering, Changchun University of Technology, Changchun, China

**Keywords:** carbon dots, Cr (VI), highly selectivity and sensitive, yellow PL emission, test papers

## Abstract

In recent years, carbon dots (CDs) are promising fluorescence probes for ions detection. In this paper, the CDs which are with an average diameter of 5.5 nm were synthesized through a simple one-step hydrothermal carbonization of ethylene diamine tetraacetic acid (EDTA) salt. The CDs have strong yellow photoluminescence (PL) with a maximum emission intensity at 550 nm under an excitation wavelength of 450 nm. As the electron transfer will occur between Cr (VI) and the CDs, yellow fluorescence was quenched after adding the Cr (VI) ions. The CDs probe allows the detection of Cr (VI) ions over a concentration range from 0 to 0.1 M (*R*^2^ = 0.987) and the lower detection limit is 10^−5^ M. Simultaneously, the CDs show highly selectivity and stability toward the detection of Cr (VI) ions.

## Introduction

In the previous several years, carbon dots (CDs) have had a wide range of applications in scientific and technological field because of their unique and novel properties (Bourlinos et al., 2012). CDs are promising as substitutes for conventional semiconductor quantum dots and organic dyes to prepare fluorescent nanosensors (Lu et al., 2017; Sun et al., 2018). In addition, CDs are superior in many aspects such as excellent water solubility, photobleaching resistance, low cytotoxicity, high biocompatibility, good photostability, tunable excitation, and adjustable emission spectrum (Baker and Baker, 2010; Qu et al., 2013; Gao et al., 2015). Due to these excellent properties, CDs can replace toxic quantum dots to apply in many clinic and environment (Goh et al., 2012; Hola et al., 2014; Gedda et al., 2016; Gong et al., 2016). Until now, there are many methods for preparing fluorescent CDs, for instance, laser ablation, arc discharge method, electrochemical oxidation, hydrothermal carbonization, ultrasonic, and microwave (Xu et al., 2011; Jia et al., 2012; Jiang et al., 2018; Liu et al., 2020). However, due to high cost, harsh synthesis conditions, complex process, low yield, and other reasons, it has certain deficiencies in achieving economic and large-scale production. It is necessary to develop a simple and economical way to synthesize carbon quantum dots. Meanwhile, it is necessary to make CDs easily exhibit quantum effects with a diameter of <10 nm (Zhao et al., 2008; Wang et al., 2011). Compared to the bulk materials, one of the most characteristic properties of CDs is the size dependence of the photophysical properties that allows their properties to be controlled simply by nanoparticle growth. As to the fluorescent probes for the detection of metal ions, CDs are promising. For example, Zhang and Chen put forward an economical and green synthetic method of fluorescent CDs and they can be applied in mercuric ion (Hg^2+^) detection on account of the induced fluorescence quenching of Hg^2+^ (Zhou et al., 2012; Zhang and Chen, 2014; Hou et al., 2015). High photoluminescent CDs has been synthesized by Zhu et al. and it has seen that in biosystems, the fluorescent carbon particles can be used for the detection of ferric ions (Fe^3+^) (Li et al., 2015). Li et al. synthesized the photoluminescent carbon dots which was employed to the detection of cobalt ions (Zhu et al., 2013). In addition, CDs are also used as fluorescent probes to detect other metal ions (Goncalves et al., 2010; Dong et al., 2012).

Chromium is widely distributed and scattered in natural environment. It is found in almost all substances. Cr (III) and Cr (VI) are the most common formations of Chromium. Cr (III) are fundamental trace elements of the body to maintain basic glucose tolerance factors and normal metabolism of lipids, proteins and fats (Zhitkovich, 2011; Xu et al., 2015). Cr (VI) has strong carcinogenicity to organisms (Krishnani et al., 2013). It can invade through the human's digestive tract, respiratory tract, skin, and mucous membranes. What's more, it may cause allergies when it is contacted with human skin. At present, as to the determination of Cr (VI) and Cr (III), many analytical techniques has been developed, and chromatography, atomic absorption spectroscopy, fluorescence quenching have been included (Arancibia et al., 2003; Kiran et al., 2008; Cai et al., 2014; Zhao et al., 2019). However, sophisticated equipment and laborious sample pretreatments are request when most of these methods are utilized. Therefore, it is necessary to design a method to detect Cr (VI) in aqueous solution directly and selectively.

**Scheme 1:**
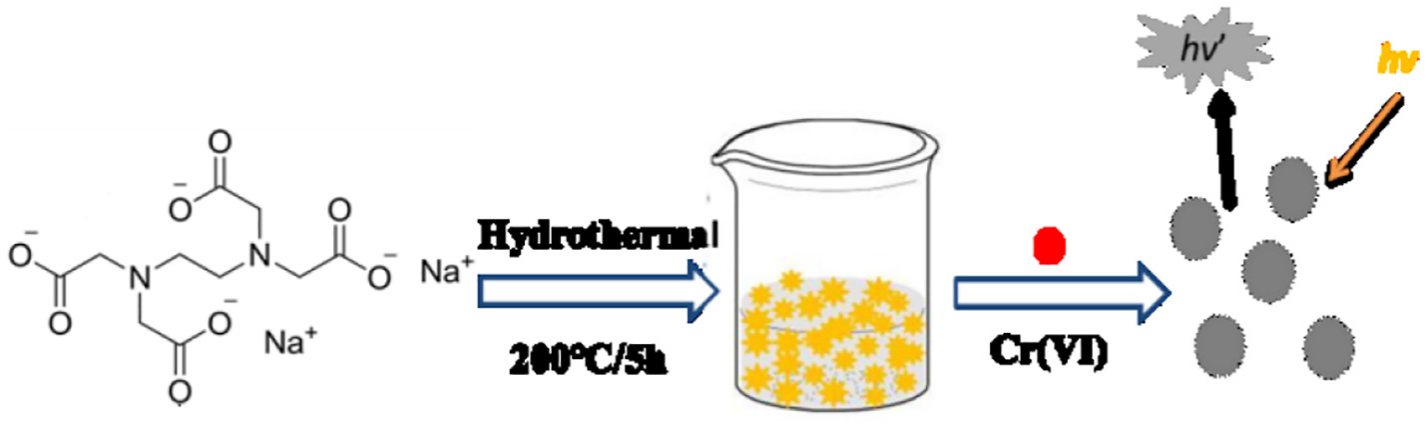
Representation of hydrothermal preparation of CDs from EDTA for the detection of Cr (VI) ions.

In this paper, we reported a plain and economical syntheses to prepare water-soluble fluorescent CDs directly from ethylene diamine tetraacetic acid (EDTA) −2Na via hydrothermal carbonization. Under the single-wavelength ultraviolet-light excitation, the strong yellow emission was emitted of the as-prepared CDs in solution. It was founded that the fluorescence intensity was decreasing with the Cr (VI)'s introduction to the CDs solution, which may ascribe the transfer of excited electrons (as shown in Scheme 1). In addition, the prepared CDs aqueous solution has high selectivity among Cr (VI), Cr (III), and other common cations. Therefore, easy-to-prepare Cr (VI) test papers was made to identify Cr (VI), which demonstrated the suitability for *in situ* on-site detection. The result provides a general guideline which can develop other semiconducting CDs to be impressive fluorescent materials in the future.

## Experimental

### Chemicals

Ethylene diamine tetraacetic acid (EDTA)-2Na, N, N-dimethylformamide (DMF), hydrochloric acid (HCl), and sodium hydroxide (NaOH) are bought from Beijing Chemical Reagent (Beijing, China). The stock solution of Cr (VI) is prepared from K_2_CrO_4_. Quinine sulfate was obtained from Sigma-Aldrich (USA). The solutions of metal ions are prepared from their nitrate, acetate, or chloride salts. All the reagents are conformed to the analytical reagent grade and are used as received originally. All aqueous solutions are prepared by making use of ultrapure deionized water from a Milli-Q purification system (Millipore, Milford, MA, United States).

### Characterization Methods

UV-Vis absorption spectrum was acquired by utilizing a spectrophotometer of TU-1901 UV-Vis (Beijing Puxi Inc., China). With a Cary Esclipse spectrofluorimeter (Varian, United States), photoluminescence (PL) experients were performed. X-ray photoelectron spectroscopy (XPS) with Mg Kα excitation (1,253.6 eV) was gathered in a VG ESCALAB MKII spectrometer (VG, Britain). Through a X-ray diffractometer system of Bede D1 high-resolution(Bede Co., United Kingdom), X-ray diffraction (XRD) spectra were recorded. Making use of a Nicolet 6700 FTIR spectrophotometer (Thermo Fisher Scientific, United States)., Fourier transform infrared (FTIR) spectroscopy was estimated at wave numbers ranging from 500 cm^−1^ to 4,000 cm^−1^. operating a JEM-2100 transmission electron microscope (TEM) at 200 kV (JEOL, Japan), resultant CDs' morphology and mean diameter were characterized. All the experiments and measurements were carried out at the room temperature under the ambient conditions.

### Synthesis of Carbon Dots

In a typical procedure, EDTA(2.64 g) was dissolved in DMF(32 mL) with stirring for 1 min. The solution of transparent aqueous was obtained under ultrasound. Afterwards, the above-mentioned mixture was transferred into a 50 mL Teflon-lined stainless-steel autoclave, then it should be reacted at 200°C for 5 h. The solution varied from transparent to brown, which indicated the carbonization of the reactant. After down to the room temperature, part of the supernatant was evaporated under vacuum at a certain temperature which aimed to remove the excessive solvent. Then, in order to dissolve the product, 20 mL of water were poured into a conical flask, and brown solution was obtained without further adjustment. Through a PTFE syringe filter, the brown supernatant was gathered and filtered with 0.22 μm pore size. The unpurified CDs exhibited yellow fluorescence under 365 nm UV lamp.

### Fluorescence Assay of Cr (VI)

For the typical Cr (VI) sensing experiments, 1 mL CDs were sequentially poured into 3.5 mL deionized water. The solution got mixed up completely and was incubated for 5 min. Then, 0.5 mL Cr (VI) standard solutions was added to give a series of different concentrations. After 10-min incubation at the room temperature, the PL spectrum of the C-dots by different excitation wavelength from 350 to 590 nm were obtained.

## Results and Discussion

### Characterization of CDs

**Figure 1:**
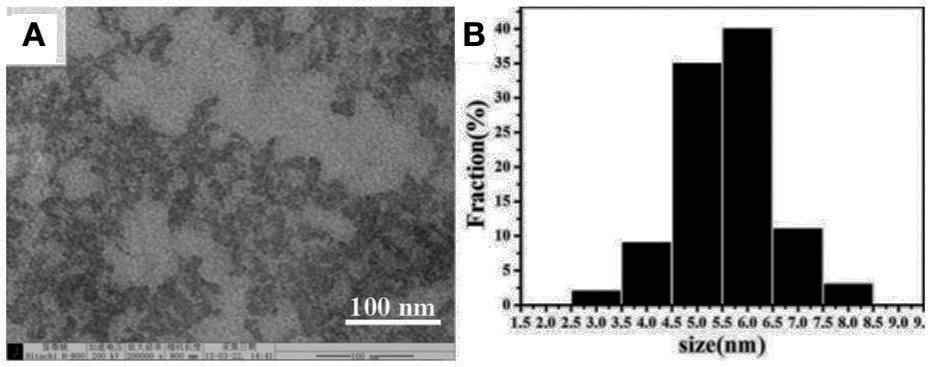
**(A)** TEM image of the synthesized CDs. **(B)** The particle size distribution of the CDs.

In order to perform the detection experiments, water-soluble CDs were prepared by hydrothermal carbonization of EDTA (Pan et al., 2010). Their morphologies were characterized by transmission electron microscopy (TEM). As it is shown in Figures 1A,B, the results suggested that the CDs are uniform and monodispersed spheres with the CDs' size that are mostly distributed in the range of 3.0–8.0 nm with the average size of 5.5 nm due to CDs' non-uniform heating and rapid formation in the rotary distiller.

**Figure 2:**
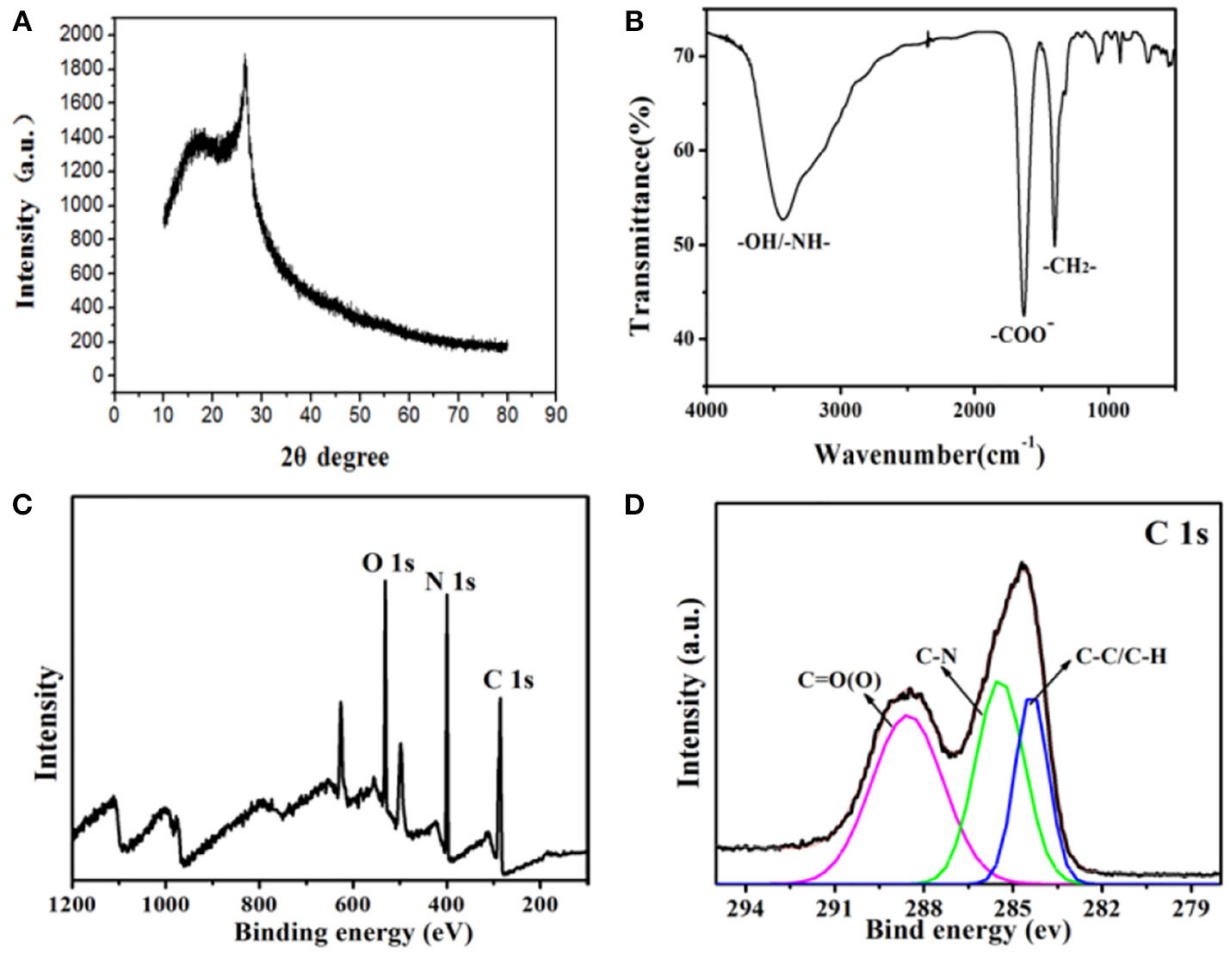
**(A)** XRD spectrum of the CDs. **(B)** FTIR spectrum of the CDs. **(C)** XPS spectrum of the as-synthesized CDs. **(D)** XPS of the C1s spectrum.

The crystal structure of the as-synthesized CDs is carried out by XRD (Figure 2A). It can be clearly observed that a broad diffraction peak at θ = 22.7° is formed, indicating that the synthesized fluorescent CDs are mainly amorphous and exist in molecular states. Then, the CDs' functional groups on surface are researched by using FTIR spectroscopy. As it is shown in Figure 2B, the CDs display a broad FTIR absorption band at 3,534 cm^−1^, which can due to the stretching vibrations of N-H and O-H. The peaks at about 1,647 cm^−1^ indicate the existence of COO-. The absorption peaks at 1,350–500 cm^−1^ belong to -CH_2_ bending vibrations. The above results are consistent with XRD, indicating that the molecular structure of EDTA exists on the surface of CDs. Moreover, these above findings verify that the as-synthesized nanoparticles are functionalized by hydroxyl, aldehyde and amino groups, which can enhance CDs' stability, and hydrophilicity in an aqueous system. X-ray photoelectron spectroscopy(XPS) has been one of the most commonly used analytical techniques to determine the elements' valence states in carbon materials. Figure 2C reveals that CDs are mainly made up of oxygen, nitrogen, and carbon with the binding energy peaks at 553.6, 402.7, and 296.4 eV, consistent with O1s, N1s, and C1s, separately. The high-resolution C1s spectrum in Figure 2D demonstrates three deconvoluted peaks at 289.1, 285.9, and 284.2 eV, which is linked to C1s states in C=O, C-N, C-C/C-H, respectively. This result is corresponding to the previous researches on carbon materials.

### Optical Properties of the CDs

**Figure 3:**
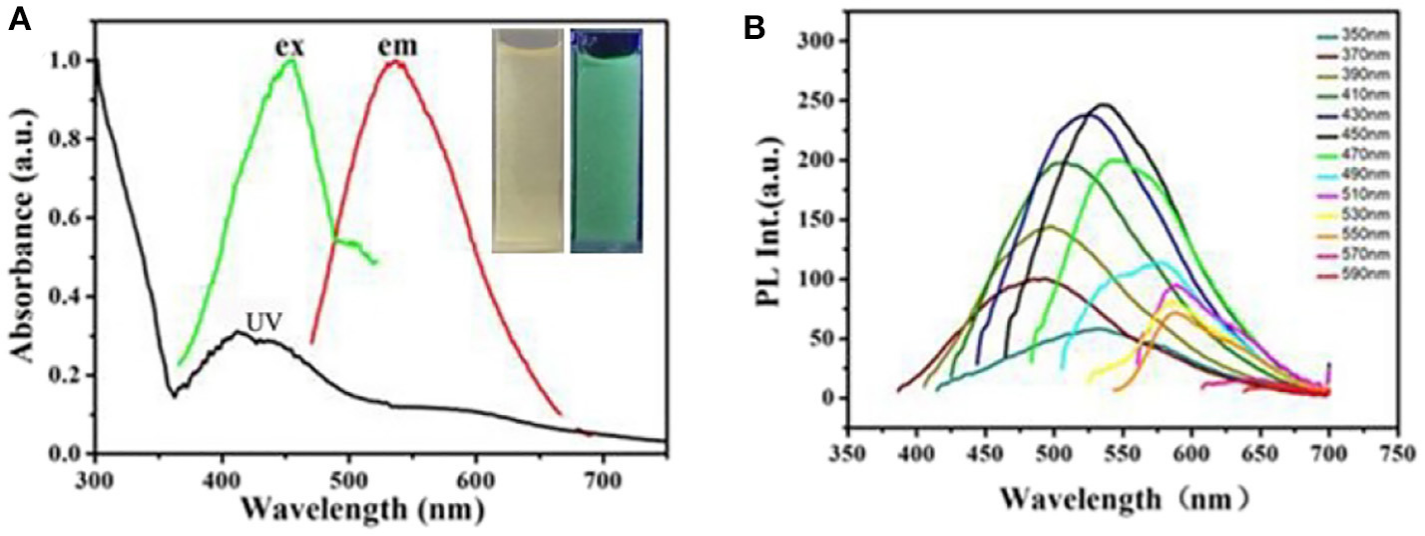
**(A)** UV-Vis absorption spectrum and fluorescent spectra of the obtained CDs. Inset: photograph of the obtained C-Dots under illumination of UV (365 nm) light. **(B)** PL emission spectra of the C-dots at different excitation wavelength from 350 to 590 nm.

As-prepared CDs' optical properties were researched by the UV–vis absorption and fluorescent spectroscopy. As what is shown in Figure 3A, the CDs' UV-Vis absorption spectrum demonstrates one absorption bands at 370 nm wavelength corresponding to n→ π^*^ (peroxide and/or epoxide groups) transitions (Zhu et al., 2014). The PL spectra display a maximum emission at 550 nm under excitation at 450 nm (Figure 3A). The photograph of CDs dispersion under the UV light of 365 nm demonstrates strong yellow emission as shown in set of Figure 3A. In addition, as what is shown, the fluorescence emission under different excitation wavelengths is studied at the same time in Figure 3B. It is clear that the PL emission peak of CDs exhibit a red-shift from nearly 450 to 550 nm with the corresponding excitation wavelength distinguish from 350 to 590 nm. This could be attributed a lot to different surface states of the as-prepared CDs (Dong et al., 2013).

**Figure 4:**
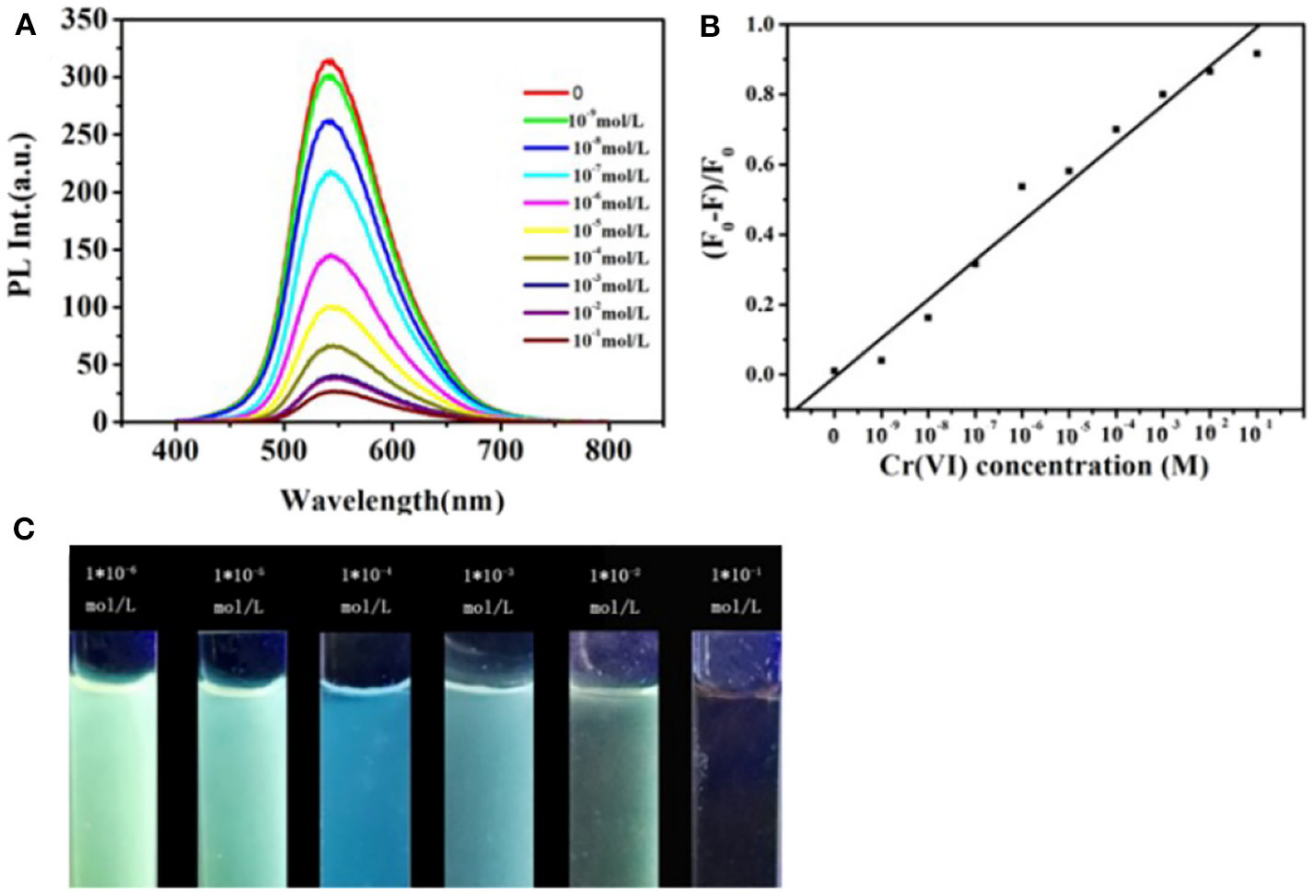
**(A)** Fluorescent emission spectra of the CDs aqueous solution with the addition of different concentrations of Cr (VI) increasing from 0 to 0.1 M, (λ_ex_ = 450 nm). **(B)** Changes of fluorescence intensity of the CDs vs. the Cr (VI) concentration (F and F0 represent fluorescence intensities with the presence and absence of metal ions, respectively). **(C)** CDs aqueous solution with the addition of different concentrations of Cr (VI) under UV light irradiation.

Figure 4 represents the fluorescent emission spectra of CDs on the increase of different concentrations of Cr (VI) ions (varying from 0 to 0.1 M). As what is shown in Figure 4A, the CDs fluorescence emission intensity at 545 nm gradually reduced with adding the concentrations of Cr (VI) ions. Figure 4B illustrates the calibration curve detection of Cr (VI) ions, which displays qualified linearity in concentration range from 0 to 0.1 M with a 0.987 correlation coefficient (the regression equation is y = 0.0576x + 0.00928). These investigating results advise that the sensing system is extremely sensitive to the Cr (VI) concentration. Correspondingly, when the Cr (VI) concentration is increased, as shown in the Figure 4C, the CDs' emission color shifted gradually from yellow, green to blue under the excitation with a 365 nm UV lamp. This indicates that sensing system is very sensitive to the Cr (VI) concentration.

### Sensitivity and Selectivity

**Figure 5:**
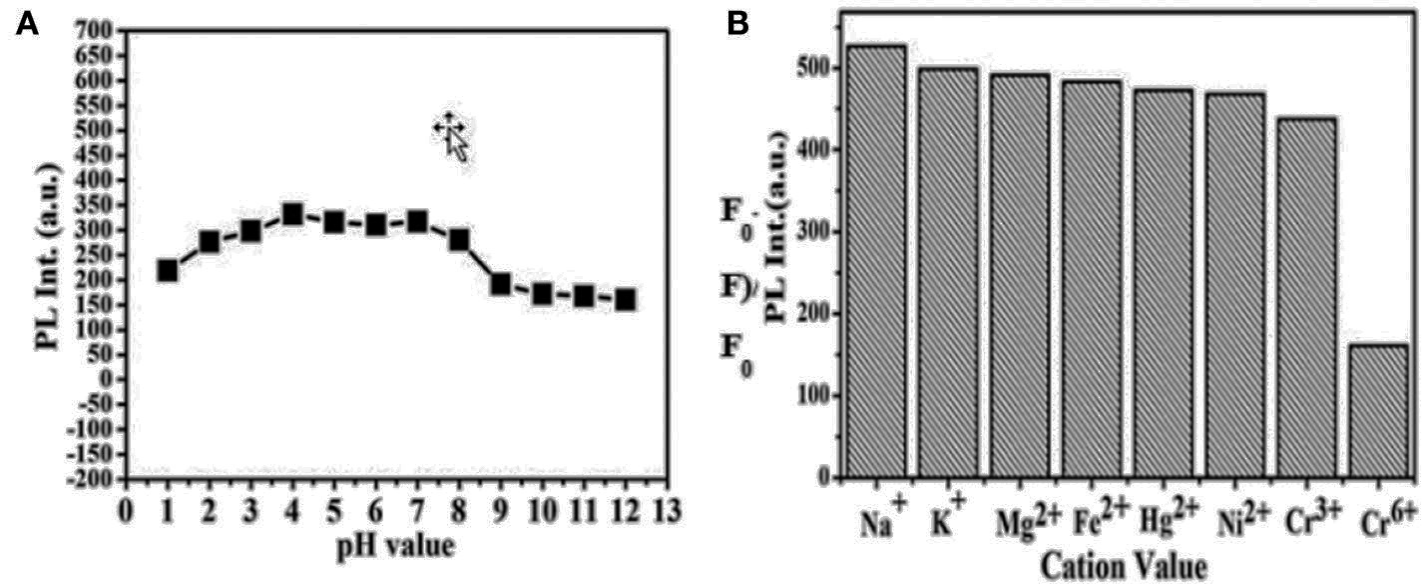
**(A)** Effect of pH on the PL intensity of CDs. **(B)** Fluorescence response of the CDs aqueous solution under the addition of different metal ions with a concentration of 10^−4^ M.

CDs' pH dependent PL behavior is studied also. It is clear in Figure 5A that CDs' PL is strong which indicates their highly stability in an extensive range of pH values (1–12). Although it is slightly growing when pH is raised from 1 to 4, this research result implies that the as-prepared CDs still exhibits qualified stability in strong acid or alkaline conditions. The CDs are applied to the detection of metal ions then. The PL intensity changes in the presence of represent active metal ions under the same conditions are examined, including Na^+^, K^+^, Ni^2+^, Hg^2+^, Fe^3+^, Cr^3+^, Cr^6+^, and Mg^2+^ (Figure 5B). The detailed UV-vis absorption spectra are presented in Figure S1. It is founded that the PL intensity of CDs was extremely quenched in the presence of Cr (VI) ions. In addition, no- tremendous quenching is founded in the presence of other metal ions, involving Cr (III) ions. It is worth noting that under the excitation of a 365 nm UV lamp, which the visual emission color of CDs solution turns from yellow to blue-green on addition of Cr (VI) ion, as what is shown in the bottom of Figure 5B. This intents, in particular Cr (III), the CDs display excellent selectivity toward Cr (VI) beyond other tested metal ions. These results implied that compared with other metal ions, the CDs can be utilized to detect Cr (VI) ions selectively in an aqueous solution. The prominent selectivity and specificity for Cr (VI) ions can be possibly belonging to it which have a stronger affinity toward the carboxylic group to CDs' surfaces.

**Figure 6:**
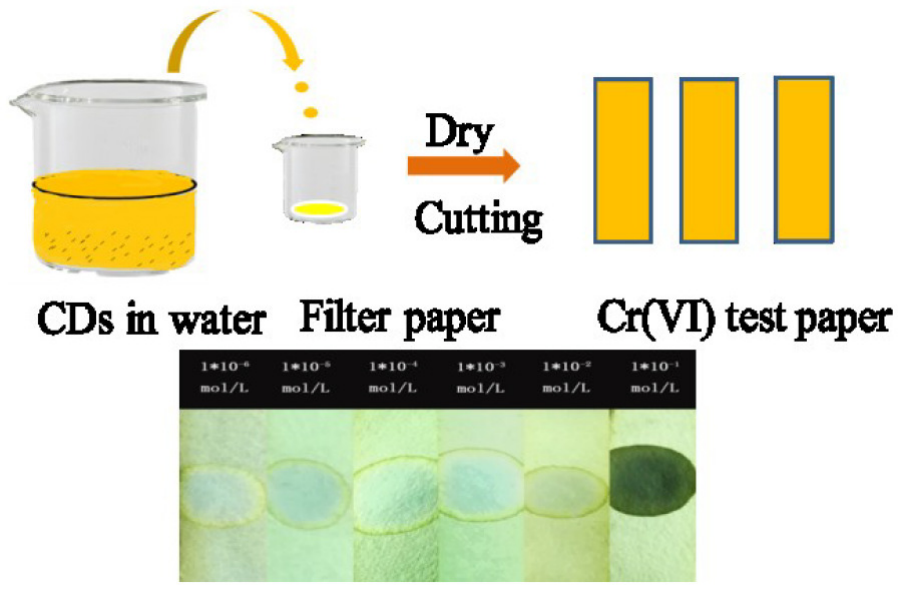
Schematic illustration of the processes for fabrication of Cr (VI) test paper (top); The bottom is the color changes of Cr(VI) test papers after dropping different concentration solutions under a 365 nm UV light.

In order to apply the platform for on-site inspection further, Cr (VI) test paper was prepared according to the procedure shown in Figure 6. In short, the concentrated CDs were dripped into the filter paper aqueous solution and dried naturally. After that, we used the test paper to visually detect Cr (VI) ions through dropping the target solution which contained Cr (VI) ions onto them. As shown in Figure 6, the color of the test paper varied from yellow to blue under the UV light of 365 nm with the increasing concentration of Cr (VI). The results show that it is promising for Cr (VI) residues in water to make *in-situ* visual detection.

## Conclusions

In summary, we have developed a easy, low-cost and eco-friendly synthesis process of CDs by making use of ordinary EDTA salt as the carbon source. Significantly, the as-prepared CDs possess excellent solubility and strong yellow fluorescence. The as-synthesized CDs were used in order to realize the sensitive and selective detection of chromium in aqueous solution and showed high sensitivity. Moreover, the decreased fluorescence intensity showed good linearity on the concentration of Cr (VI) ions in aqueous solution and the limitation of detection could reach as low as 10^−5^ M. We expect his strategy may provide a new approach for bio-imaging, bio-sensors and environmental applications.

## Data Availability Statement

The original contributions presented in the study are included in the article/Supplementary Materials, further inquiries can be directed to the corresponding author/s.

## Author Contributions

YY designed, analyzed data, and wrote the main manuscript. XC performed the experiments and performance evaluation. YW and MW conceptualized, designed, and edited the main manuscript. YM and XY helped design complement experiments and reviewed and edited the manuscript. All authors contributed to the review and approval of the manuscript.

## Conflict of Interest

The authors declare that the research was conducted in the absence of any commercial or financial relationships that could be construed as a potential conflict of interest.
