# Supplementary material for: A Novel Fluorescent Test Papers Based on Carbon Dots for Selective and Sensitive Detection of Cr (VI)
Source: Front Chem. 2020 Dec 1;8:595628. doi: 10.3389/fchem.2020.595628 (PMC7736037; doi:10.3389/fchem.2020.595628)
Supplement: Supplementary file 1 [file Image_1.pdf]

Supplementary Material

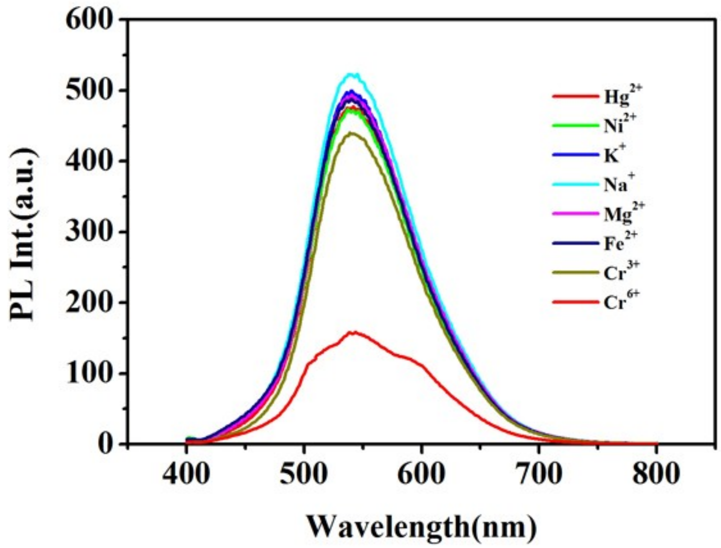

Fig.S1 Fluorescent emission spectra of the C-dot probe toward Cd^{6+} ions against other metal ions. The concentration of each of the metal ions was 10^{-4} M.
